# Help‐Seeking and Substance Use Among Police Staff After the 2018 Strasbourg Christmas Market Attack

**DOI:** 10.1002/ajim.70092

**Published:** 2026-05-24

**Authors:** Nathalie Nourry, Ludivine Nohales, Laurence Lalanne, Emmanuel Fort, Maria Gonzalez, Pierre Vidailhet, Amaury C. Mengin, Barbara Charbotel

**Affiliations:** ^1^ Service de Pathologies Professionnelles et Médecine du Travail Hôpitaux Universitaires de Strasbourg Strasbourg France; ^2^ Faculty of Medicine Strasbourg University Strasbourg France; ^3^ Université de Lyon, Université Claude Bernard Lyon 1, Université Gustave Eiffel, UMRESTTE Lyon Cedex France; ^4^ SMST‐CRPPE Lyon (Centre Régional de Pathologies Professionnelles et Environnementales), Hospices Civils de Lyon Lyon France; ^5^ Psychiatry, Mental Health and Addictology Department Strasbourg University Hospital Strasbourg France; ^6^ INSERM U1329, Strasbourg Translational NEuroscience and Psychiatry (STEP), Team Psychiatry Strasbourg France; ^7^ Fédération de Médecine Translationnelle de Strasbourg France; ^8^ Regional Center for Psychotraumatism Great East Strasbourg France

**Keywords:** alcohol use, depression, mental health service, police force, post‐traumatic stress disorders, terrorism

## Abstract

**Background:**

The use of mental health services by police staff is usually low. After the 2018 attack on the Strasbourg Christmas market, police officers exposed to psychotraumatic risks were found to have a higher PTSD risk. This study aims to describe the help‐seeking and substance use by police staff after the attack.

**Methods:**

A questionnaire was sent to 1697 police officers 3 months after the attack. The 475 respondents were classified as exposed (took action to stop the terrorists, pursued them in the city, or helped a victim) or unexposed and indirect‐exposed. Counselling, treatment, alcohol and tobacco consumption, and their habitus changes were assessed. Univariable analysis was followed by multivariable analysis adjusted for gender, age, number of previous traumatic events, level of exposure, and probable PTSD or depression, using logistic regression.

**Results:**

Of the 475 respondents, 10% consulted a psychologist/psychiatrist, 24% a physiotherapist and 62% their general practitioner within 3 months of the assault. Initiation of new anxiolytic/antidepressant treatment (OR = 4.59 [1.11–18.91]), consultation with a psychologist/psychiatrist (OR = 5.02 [2.44–10.32]), increased alcohol or tobacco consumption (OR = 10.42 [3.45–31.43]) were associated with probable PTSD.

**Conclusions:**

The use of mental health services is insufficient for the care of probable PTSD, which is associated with increased tobacco or alcohol use, encouraging further research to improve the use of mental health services among police staff.

## 1 Introduction

The barriers to seeking mental health care in the general population are well known [1]. The main ones are lack of awareness of the need for care and the fear of stigma. Previous studies showed that mental help‐seeking was correlated with symptom severity and the presence of post‐traumatic stress disorder (PTSD) among civilians exposed to a terrorist attack [2]. However, among police staff, specific barriers are described in the literature [3] and may hinder mental health‐seeking in this population [4, 5, 6, 7, 8, 9]. These factors include higher mental health stigma, worries about confidentiality, male gender, younger age, and fewer years of service, as well as a lack of training in mental health. According to previous research, mental health training is often provided to reduce stigma toward service users and fosters changes in police officers' behaviour regarding their own mental health or that of their colleagues. Other factors, such as police culture—notably, hypermasculine attitudes, an exaggerated belief in one's self‐efficacy, the idea that police officers are there to help the public rather than be helped, and the fear of the impact on their careers— may lead to the adoption of inappropriate coping strategies [8, 10].

Members of the police force generally suffer psychological trauma in the course of their daily work or during extremely violent situations such as natural disasters or terrorist attacks [11, 12, 13, 14, 15]. They are also exposed to stress caused by the organisational work environment. Whether it be mass events, routine exposure to psychological trauma, or organisational stress factors, all these factors can be sources of PTSD, according to the review by Sherwood et al. [16]. Depression is more often linked to organisational professional factors [16]. Nevertheless, depression is often cited as a comorbidity of PTSD [17]. Police officers are therefore likely to develop post‐traumatic stress disorder or depression and are therefore in need of mental health care, especially since they have easy access to a weapon.

The way to treat post‐traumatic stress disorder is set out in international recommendations in England and Australia [18, 19]. French recommendations are currently being drawn up. Psychotherapy is the recommended first‐line treatment, particularly Trauma‐Focused Cognitive and Behavioural Therapy (TF‐CBT) and Eye Movement Desensitization and Reprocessing (EMDR). Background psychotropic medication is indicated as a second‐line option.

The 2018 attack on the Strasbourg Christmas market in France and the 2‐day hunt for the terrorists involved many police officers. Their exposure varied from dealing with deceased victims to exchanging gunfire with the suspected terrorist, who was subsequently shot dead by a police patrol, as well as contacting victims' families and interviewing victims and witnesses. No physical injuries were reported among the police officers.

Medical and psychological support units were set up for civilians in the immediate aftermath of the event [2]. Police staff were able to receive support from in‐house psychologists and medical examinations by occupational physicians. Information about the possibility of receiving psychological support was disseminated through the hierarchy or through outreach work within the departments. Some police staff also made use of the general healthcare services. After three weeks, the special arrangements were discontinued and the usual range of healthcare services returned.

Excessive alcohol consumption is of concern, with an estimated 5% of the police community dependent on alcohol and 25.7% engaged in hazardous drinking [20]. A link has been described between substance abuse, including alcohol, and the existence of PTSD [21]. Alcohol and other substance use are coping mechanisms for PTSD [21, 22].

In France, as elsewhere, there is a need for more knowledge about the care of victims, especially police staff, after a major event such as an attack. Following the Utoya attack in Norway in 2011, more survivors, especially women, sought care from general practitioners and psychiatrists than in the previous year. Sixty‐nine percent of the population received mental health care [23]. A study of healthcare use among civilians 8–11 months after the Paris attacks in November 2015 found that 56% of victims with PTSD or depression consulted a psychiatrist or psychologist, and 33% consulted their general practitioner [3]. Care‐seeking among first responders, including police officers, nurses, firefighters and civil protection volunteers, was also studied 8–11 months after the Paris attacks; 38% of first responders suffering from complete or partial PTSD or depression sought mental health care. No significant differences were observed among the various professions represented among first responders regarding post‐immediate support, but a significant difference was found for immediate support: police personnel sought immediate care less frequently than firefighters [24]. After the Strasbourg attack, 86% of civilians with probable PTSD consulted a mental health professional and 30% received psycho‐medical treatment [2]. There has been no research into the use of mental health services by police staff.

The aim of this study is to describe the use of healthcare and changes in alcohol and tobacco consumption in this population, that is, police officers. Using the example of the terrorist attack on the Christmas market in Strasbourg, France, in December 2018, the study examines police officers' use of healthcare services and changes in their alcohol and tobacco consumption during the three months following the attack. Two hypotheses were put forward: (1) after an event such as the Strasbourg attack, police officers made insufficient use of mental health care; (2) police officers with probable PTSD or depression consumed more alcohol and tobacco since the attack than other police officers.

## 2 Materials and Methods

**Figure 1:**
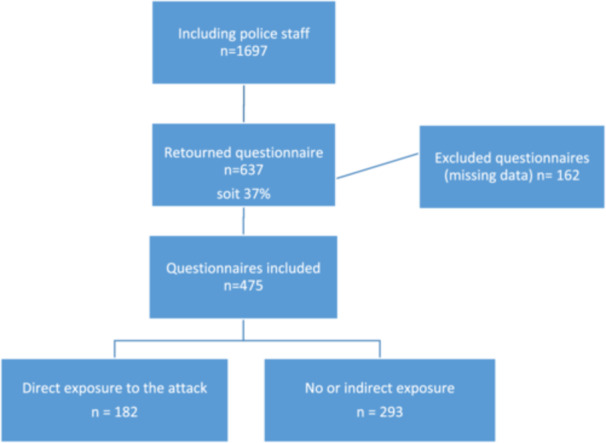
Flowchart.

This study was conducted by the occupational health service among police staff between March and April 2019, three months after the Strasbourg Christmas market attack (which occurred on December 11, 2018). All active police, technical, scientific, administrative, and logistical personnel were included (see flow chart Figure 1). After the collective and individual information, employees were invited by email to their professional inbox to complete a self‐questionnaire online or on paper, depending on the service. Further details pertaining to the study methodology can be found in a previous article [25].

### 2.1 Measures

#### 2.1.1 Socio‐Demographic Characteristics

The following socio‐demographic characteristics were collected: age, gender, marital status (living alone or in a couple), level of education in 3 classes (less than hight school, high school graduate, post‐secondary) and occupational characteristics: job (administrative, technical and forensic, police officer assistant, police officer, police inspector, and police commissioner) (Table 1).

**Table 1:** Sociodemographic, exposure level, and medical characteristics of the population.

	*N* or Mean (sd) *n* = 475	%
Age	42 years (9.14)	
Gender		
Female	127	27
Male	348	73
Marital status		
Living alone	111	23
Living with a partner	362	77
Level of education		
Less than high school	47	10
High school graduate	245	52
Post‐secondary	179	38
Job		
Admin, technical and forensic	71	15
Police officer assitant	21	4
Police officer	330	69
Police inspector	42	9
Police commissioner	11	2
Exposure to attack		
No exposure	212	45
Indirect exposure	81	17
Direct exposure	182	38
No exposure+ indirect exposure	293	62
Direct exposure	182	38
Number of previous traumatic events (LEC)		
< 6	116	24
6 to < 9	94	20
9 to < 12	129	27
≥ 12	23	29
Probable PTSD		
Yes	58	12
No	417	88
Depression		
Yes	56	12
	419	88

#### 2.1.2 Exposure

Exposure during the attack was assessed using 15 questions, which classified participants into three levels according to DSM‐5 criteria A: unexposed (not having been at work during the attack and the pursuit), indirectly exposed (criterion A item 3: contact with anyone directly threatened or injured by the terrorist, or search for the terrorist out of city center), and directly exposed (criterion A items 1, 2, or 4: any intervention on the spot of attack, or pursuit of the terrorist in the city center, contact with the terrorist or rescue action on a victim). Questions are provided in the supplementary material (Table S1). This study uses a binary variable to group unexposed and indirectly exposed participants on one side and directly exposed participants on the other, as probable PTSD has been shown to be linked to direct exposure alone [25].

Previous traumatic events were also assessed using the Life Events Checklist for DSM‐5 (LEC‐5). The total number of potentially traumatic events reported by survey respondents was counted, regardless of whether they were direct or indirect victims. The number of events reported ranged from 0 to 17. Four categories were defined based on quartiles (below 6, 6 to over 9, 9 to over 12, and 12 or over) [26].

### 2.2 Mental Health

#### 2.2.1 Post‐Traumatic Stress Disorder

Post‐traumatic stress disorder was assessed using the 20‐item PCL ‐5 scale with reference to the attack on December 11, 2018 [26, 27]. Each item is rated using a Likert scale from (0) “not at all” to (4) “extreme”. The items assess the four DSM‐5 clinical symptom clusters: Cluster B, symptoms of reliving (item 1–5), Cluster C, symptoms of avoidance (items 6–7), Cluster D, symptoms of altered cognition and mood (8–14), Cluster E, symptoms of hypervigilance (items 15–20). According to DSM‐5 rules, a symptom is considered to be present if an item is rated two or more. For a diagnosis of full‐blown PTSD, the person must present at least one clinical sign from group B, 1 from group C and two signs from groups D and E [28]. A partial PTSD diagnosis requires at least two or three clinical signs from symptom groups B, C, D, or E [29]. Cronbach's *α* in our sample was 0.95. In our study, we grouped partial and full PTSD together. As our aim was to assess the use of treatment, we considered that cases of partial post‐traumatic stress disorder among police officers required at least some form of follow‐up and should therefore also be treated.

#### 2.2.2 Depression

The presence of depression was assessed using the validated PHQ‐9 questionnaire. It consists of 9 questions that allow a diagnosis of depression and an assessment of its severity. Answers to the questions range from 0 to 3, and the total score ranges from 0 to 27 [30]. A 2‐class variable was created, with no depression and mild depression on the one hand, and moderate, moderate to severe, and severe depression on the other, i.e. a cut‐off greater than or equal to 10. The Cronbach's *α* in our sample was 0.86.

##### 2.2.2.1 Use of Medical Care

We asked police officers about treatment received 3 months after the event, without specifying dates or names.

#### 2.2.3 Consultation With General Practitioner

Agents were asked: “When was the last time you had a consultation (office visit, home visit, or telephone consultation) with a general practitioner (GP) or your own GP?” The possible answers were: “less than 4 weeks”, “between 4 weeks and less than 3 months” and “more than 3 months”. Responses were grouped as follows: more than 3 months vs. less than 3 months (i.e., before or after the attack).

#### 2.2.4 Consultations With Psychiatrist and/or Psychologist

The same question was asked about consultations with a psychiatrist or psychologist: “Have you consulted a psychologist, psychotherapist, or psychiatrist in the last 3 months?” The response was binary: “yes” or “no”.

#### 2.2.5 Consultations With a Physiotherapist

The question posed to staff enquired whether they had consulted a physiotherapist within the preceding 3 months. The answer had to be “yes” or “no”.

#### 2.2.6 Treatment Evaluation

The treatments taken were evaluated by four questions. The first question was about self‐medication (a). The second question was about the prescription of a treatment by a doctor (b). The third question was about the prescription of an antidepressant or anxiolytic (c). The last question was about the prescription of a hypnotic (d).

(a,b) Self‐medication and prescription drugs were assessed by two closed questions, the questions related to the last 2 weeks before completing the questionnaire and were respectively formulated as follows: “Did you take any medicines, herbal remedies or vitamins not prescribed by a doctor”, “Did you take any medicines prescribed by a doctor (excluding the pill)”, the possible answers were “yes” or “no”.

(c,d) For the evaluation of antidepressant or anxiolytic treatment, and for treatment with sleeping pills the possible answers were: “no”, “yes, for less than 3 weeks”, “yes, for 3 to 12 weeks”, “yes, for 3 to 6 months” and “yes, for more than 6 months”. Grouping was done to have a variable with the following 3 classes: (0) “no”, (1) “yes, prior to the event”, which includes “for more than 6 months” and “for 3 months and less than 6 months”, (2) “yes, since the event”, which includes “yes for 3 to 12 weeks” and “yes for less than 3 weeks”.

##### 2.2.6.1 Care Summary

Finally, we defined a variable for the use of care with three categories: the first: no care, no physiotherapy, no self‐medication, no general practitioner, no psychologist/psychiatrist, no antidepressant/anxiety or hypnotic treatment, the second: care, but no psychotropic treatment or mental health follow‐up and finally the third: people who have at least one follow‐up with a mental health specialist or psychotropic treatment.

##### 2.2.6.2 Alcohol and Tobacco

Police staff may be asked to answer a questionnaire which could reveal pathological use of the substance, particularly alcohol and put them in difficult position. As a result, they might refuse to answer. For this reason, to assess alcohol and tobacco consumption, we preferred to question medically their use and to quote responses as following than to assess their use with validated questionnaires.

#### 2.2.7 Alcohol Consumption

Alcohol consumption was assessed by the following question: “How often do you drink alcohol (beer, wine, alcopops, hard liquor)?” The five possible frequency responses were: (0) “never”, (1) “once a month or less”, (2) “several times a month”, (3) “several times a week”, (4) “at least four times a week”. A binary variable was created by grouping “never” and “once a month or less” and “several times a month” under the heading “low frequency of drinking” and “several times a week” and “at least four times a week” under the heading “excessive frequency of drinking”.

#### 2.2.8 Tobacco Consumption

At the time of the survey, respondents indicated whether they were “smokers”, “ex‐smokers (quit at least a year ago)” or “non‐smokers”.

#### 2.2.9 Changes in Alcohol and Tobacco Consumption

For both types of consumption, we asked whether their respective consumption had changed since the attack. Three answers were possible: “I have not changed my consumption”, “I have reduced my consumption” and “I have increased my consumption”. The answers “I have not changed my consumption” for alcohol or tobacco and “I have reduced my consumption” for alcohol or tobacco were grouped together vs. “I have increased my consumption”.

### 2.3 Ethics

The study was approved by the Comité d'Ethique pour la Recherche, the Ethics Committee of the Faculties of Medicine, Odontology, Pharmacy, Nursing, Physiotherapy, and Maieutics, and the University Hospitals of Strasbourg, France (CE‐2019‐17). Prior to participation, all subjects received written information detailing the objectives of the study. Written consent was obtained from all participants. The anonymised data collected for this research study are not linked to any personally identifiable information.

### 2.4 Statistical Analysis

After describing the different care items, alcohol and tobacco consumption, the difference between respondents with probable PTSD (complete or partial) vs. without PTSD and between respondents with depression vs. without depression was analysed using *χ* ^2^ or Fisher's exact test.

The use of health care and the use of tobacco and alcohol were analysed in relation to socio‐demographic, occupational, and medical characteristics.

The differences for the indicators of health care consumption and tobacco and alcohol consumption were first analysed by exposure to the assault without adjustment and expressed as odds ratios with their confidence intervals. Multivariable logistic regression was then performed, first adjusting for exposure to the attack, gender, and then adding either the presence of probable PTSD or depression. The interaction between age and gender was also tested. Regardless of the type of healthcare utilization, the interaction between age and gender was not significant. A second set of models was developed, adjusted for age and the number of previous traumatic events to check that this would not influence our results.

The variance inflation factor (VIF) was estimated for the set of covariates for each model.

The statistical significance level for all analyses was set at 0.05. All analyses were performed using R 4.3.1.

## 3 Results

Of the 1697 national police staff in the Strasbourg region who received the questionnaire, 637 responded (37%). In the end, 475 questionnaires were analysed. 163 contained missing data from which the variables of interest had to be excluded (see flowchart Figure 1).

The main characteristics of the respondents are shown in Table 1. The population is predominantly male (75%) with an average age of 42 years. The vast majority were police officers with a baccalaureate level of education (52%). At the time of the attack, 38% were considered to have been directly exposed, 17% indirectly exposed, and 45% not exposed. Regarding the number of previous traumatic events, only 15 participants had experienced none; on average, the participants reported 8.80 events (standard deviation = 4.39).

The study population included 58 respondents (12%) with symptoms of complete or partial PTSD and 56 respondents (12%) with depression. In addition, 15 respondents (3%) had suicidal ideation. It is of interest that some respondents had multiple disorders, including probable PTSD and depression (*n* = 27, 5.7%).

For the population, healthcare consumption is described in Table 2.

**Table 2:** The use of healthcare services, smoking, and alcoholism among the whole population, and among those with probable post‐traumatic stress disorder and depression.

	Total	Probable PTSD *n* (%)			Depression *n* (%)			
Type of mental health care	*n* (%)	Yes	No	*p*	Yes		No	*p*
Consultation with a GP								
Yes	293 (62)	41 (72)	252 (61)	0.11	39 (71)		254 (61)	0.16
No	178 (38)	16 (28)	162 (39)		16 (29)		162 (39)	
Consultation with a mental health professional (psychiatrist or psychologist)								
Yes	45 (10)	19 (33)	26 (6)	< 0.0001	16 (29)		29 (7)	< 0.0001
No	427 (90)	39 (67)	388 (94)		40 (71)		387 (93)	
Physiotherapist								
Yes	114 (24)	18 (32)	96 (23)	0.17	18 (33)		96(23)	0.12
No	355 (76)	39 (68)	316 (77)		37 (67)		318 (77)	
Automedication								
Yes	135 (29)	22 (38)	113 (27)	0.09	33 (59)		102 (24)	< 0.0001
No	337 (71)	36 (62)	301 (73)		23 (41)		314 (76)	
Medication prescribed by a doctor								
Yes	159 (33)	20 (36)	139 (34)	0.89	31 (55)		128 (31)	0.0003
No	313 (66	38 (65)	275 (66)		25 (45)		288 (69)	
Antidepressant treatment								
Yes, but before the event	14 (3)	4 (7)	10 (2)	0.02	7 (13)		7 (2)	0.0001
Yes, since the event	7 (1)	3 (5))	4 (1)		3 (5)		4 (1)	
No	451 (96)	51 (88)	400 (97)		46 (82)		405 (97)	
Hypnotic treatment								
Yes, but before the event	7 (1)	0	7 (2)	0.10	4 (7)		3 (1)	0.0008
Yes, since the event	3 (1)	2 (3)	1 (0)		2 (4)		1 (0)	
No	462 (98)	56 (97)	406 (98)		50 (89)		412 (99)	
Summary								
No	172 (36)	15 (26)	157 (38)	< 0.0001	15 (27)		157 (38)	< 0.0001
No specialized care	244 (52)	23 (40)	221 (53)		21 (37)		223 (54)	
Specialized care	56 (12)	20 (36)	36 (9)		20 (36)		36 (8)	
Tobacco								
Ex‐smoker	73 (16)	8 (14)	65 (16)	0.97	8 (14)		65 (16)	0.80
Smoker	96 (20)	12 (21)	84 (20)		14 (25)		82 (20)	
Non smoking	302 (64)	38 (65)	264 (64)		34 (61)		268 (64)	
Frequency of alcohol consumption								
Excessive frequency	168 (36)	25 (43)	143 (35)	0.20	21 (37)		147 (35)	0.75
Low frequency	304 (64)	33 (57)	271 (65)		35 (63)		269 (65)	
Increased alcohol and/or tobacco consumption								
Yes	16 (3)	10 (18)	6 (2)	< 0.0001	4 (8)		12 (3)	0.10
No	439 (97)	45 (82)	394 (98)		49 (92)		390 (97)	

In the last 3 months, 62% of participants had consulted their general practitioner, 10% had consulted a psychologist or psychiatrist, and 24% had consulted a physiotherapist. Regarding treatment, 29% were self‐medicating and 33% were taking medication prescribed by a doctor. Very few had taken antidepressants or anxiolytics (*n* = 7 or 1%) or hypnotics (*n* = 3 or 1%) since the attack. In summary, 36% had received no treatment at all, half had been treated by their GP alone, and 12% had received specialist mental health care.

Of the respondents with probable PTSD (*n* = 58), a third had consulted a psychiatrist or psychologist (*n* = 19) and 7% (*n* = 4) had started treatment with an antidepressant, anxiolytic, or hypnotic.

Among those with depression (*n* = 56), 29% (*n* = 16) had begun mental specialist follow‐up and 7% (*n* = 4) psychotropic drug treatment.

**Figure 2:**
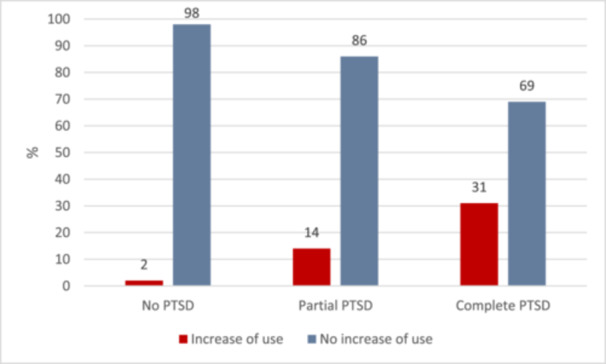
Increase in alcohol or tobacco use according to level of severity of probable PTSD, *n* = 475, *p* < 0.0001.

**Figure 3:**
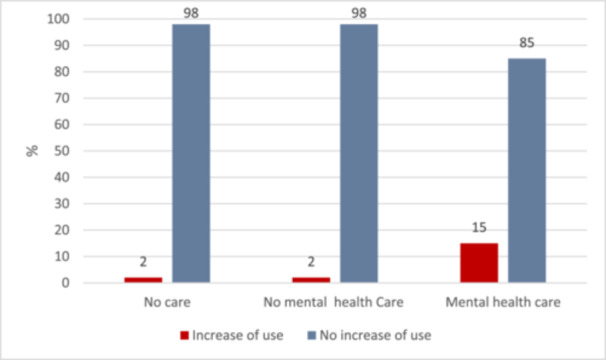
Increase in alcohol or tobacco use according to level of care seeking *n* = 475, *p* = 0.0003.

Excessive alcohol consumption affects 36% of our sample. The use of alcohol or tobacco increases with the severity of probable PTSD, but also with the use of care, as shown in Figures 2 and 3.

The factors associated with seeking mental healthcare were examined using different models (see Table 3).

**Table 3:** Results of multivariable analysis of healthcare, alcohol, and tobacco use and trends.

	Independant variable	Models adjusted for exposure	Models adjusted for exposure and gender	Models adjusted for exposure, gender and probable PTSD	Models adjusted for exposure, gender and depression
General practitioner	Exposure *ref = no or indirect exposure*	0.91 [0.62–1.34] *p* = 0.64	1.00 [0.68–1.48] *p* = 0.98	0.95 [0.64–1.42] *p* = 0.82	1.00 [0.67–1.48] *p* = 0.99
	Gender *ref = male*	/	**1.84 [1.17–2.89] *p*** = **0.008**	**1.75 [1.11–2.77] *p*** = **0.02**	**1.77 [1.12–2.80] *p*** = **0.015**
	Probable PTSD *ref= no PTSD*	/	/	1.51 [0.80–2.84] *p* = 0.20	/
	Depression ref = no	/	/	/	1.34 [0.72–2.52] *p* = 0.36
Physiotherapist	Exposure *ref = no or indirect exposure*	1.35 [0.88–2.07] *p* = 0.17	1.40 [0.91–2.18] *p* = 0.13	1.34 [0.86–2.10] *p* = 0.20	1.40 [0.90–2.16] *p* = 0.14
	Gender *ref = male*	/	1.25 [0.77–2.01] *p* = 0.36	1.19 [0.73–1.95] *p* = 0.47	1.17 [0.72–1.91] *p* = 0.52
	Probable PTSD *ref= no PTSD*	/	/	1.36 [0.73–2.55] *p* = 0.33	/
	Depression ref = no	/	/	/	1.55 [0.83–2.89] *p* = 0.16
Consultation with a psychiatrist or psychologist	Exposure *ref = no or indirect exposure*	**2.19 [1.18–4.08] *p*** = **0.013**	**3.33 [1.69–6.55] *p*** = **0.0005**	**2.50 [1.22–5.11] *p*** = **0.012**	**3.61 [1.78–7.35] *p*** = **0.0004**
	Gender *ref = male*	/	**5.52 [2.81–10.87] *p*** < **0.0001**	**4.44 [2.19–9.00 *p*** = **< 0.0001**	**4.73 [2.33–9.59] *p*** = **< 0.0001**
	Probable PTSD *ref= no PTSD*	/	/	**5.02 [2.44–10.32] *p*** < **0.0001**	/
	Depression ref = no	/	/	/	**4.34 [2.08–9.03] *p*** < **0.0001**
Self‐medication	Exposure *ref = no or indirect exposure*	0.75 [0.49–1.14] *p* = 0.17	0.87 | 0.56–1.33] *p* = 0.51	0.81 [0.52–1.27] *p* = 0.37	0.83 [0.54–1.30] *p* = 0.42
	Gender *ref = male*		**2.39 [1.54–3.70] *p*** = **0.0001**	**2.27 [1.45–3.55] *p*** = **0.0003**	**2.01 [1.27–3.18] *p*** = **0.003**
	Probable PTSD *ref= no PTSD*			1.48 [0.81–2.72] *p* = 0.20	
	Depression ref = no				**3.81 [2.11–6.90] *p*** < **0.0001**
Prescription drugs	Exposure *ref = no or indirect exposure*	0.94 [0.63–1.39] *p* = 0.74	1.03 [0.69–1.55] *p* = 0.87	1.05 [0.69–1.58] *p* = 0.82	1.02 [0.67–1.53] *p* = 0.94
	Gender *ref = male*		**1.81 [1.18–2.78] *p*** = **0.006**	**1.84 [1.19–2.84] *p*** = **0.006**	**1.60 [1.03–2.49] *p*** = **0.04**
	Probable PTSD *ref= no PTSD*			0.91 [0.50–1.67]*p* = 0.77	
	Depression ref = no				**2.49 [1.39–4.44] *p*** = **0.002**
New “psy” treatment since attack n = 9	Exposure *ref = no or indirect exposure*	**5.87 [1.20–28.56] *p*** = **0.03**	**5.99 [1.20–29.89] *p*** = **0.03**	4.38 [0.84–22.73] *p* = 0.08	**5.86 [1.13–30.48] *p*** = **0.03**
	Gender *ref = male*		1.13 [0.22–5.73] *p* = 0.88	0.80 [0.15–4.25] *p* = 0.79	0.82 [0.15–4.51] *p* = 0.82
	Probable PTSD *ref= no PTSD*			**4.59 [1.11–18.91] *p*** = **0.03**	
	Depression ref = no				**6.90 [1.71–27.79] *p*** = **0.007**
Treatment started before the attack	Exposure *ref = no or indirect exposure*	0.57 [0.20–1.60] *p* = 0.28	0.74 [0.25–2.14] *p* = 0.57	0.67 [0.22–2.00] *p* = 0.47	0.72 [0.24–2.17] *p* = 0.56
	Gender *ref = male*		**3.82 [1.47–9.91] *p*** = **0.006**	**3.54 [1.34–9.36] *p*** = **0.01**	2.65 [0.96–7.32] *p* = 0.06
	Probable Ptsd *ref= no PTSD*			1.69 [0.50–5.62] *p* = 0.39	
	Depression ref = no				**6.03 [2.24–16.23] *p*** = **0.0004**
**Modif alcool tabac 2cl**	Exposure *ref = no or indirect exposure*	**5.23 [1.66–16.49] *p*** = **0.005**	**6.31 [1.94–20.57] *p*** = **0.002**	**3.92 [1.14–13.49] *p*** = **0.03**	**6.56 [1.98–21.76] *p*** = **0.002**
	Gender *ref = male*	/	2.48 [0.84–7.32] *p* = 0.10	1.57 [0.49–5.00] *p* = 0.44	2.28 [0.76–6.88] *p* = 0.14
	Probable PTSD *ref= no PTSD*	/	/	**10.42 [3.45–31.43] p** < **0.0001**	/
	Depression ref = no	/	/	/	2.55 [0.76–8.55] *p* = 0.13

*Note:* Bold values indicate *p*‐value inferior at 0.05.

Starting a new anxiolytic/antidepressant treatment (OR = 4.59 [1.11–18.91] *p* = 0.03), consulting a psychologist or psychiatrist (OR = 5.02 [2.44–10.32] *p* < 0.0001) and increasing alcohol or tobacco consumption (OR = 10.42 [3.45–31.43] *p* < 0.0001) were significantly associated with the presence of probable PTSD, after adjustment for gender and level of exposure.

Consultation with a psychologist or psychiatrist (OR = 4.34 [2.08–9.03] *p* < 0.0001), self‐medication (OR = 3.81 [2.11–6.90] *p* < 0.0001), prescribing non‐specific medication (OR = 2.49 [1.39–4.44] *p* = 0.002), starting a new anxiolytic/antidepressant treatment (6. 90 [1.71–27.79] *p* = 0.007), and treatment prior to the attack (OR = 6.03 [2.24–16.23] *p* = 0.0004) are significantly linked to the presence of depression after adjustment for gender and level of exposure.

Depending on the level of exposure, the respondents' use of health care differed. Results show a significant difference, with more consultations with a psychiatrist or psychologist, more initiation of antidepressant or anxiolytic treatment, and more changes in alcohol or tobacco consumption among those directly exposed (regardless of the pathology present).

Female gender was also associated with a significant increase in the use of healthcare services, such as consultations with a general practitioner, psychiatrist or psychologist, self‐medication, prescription of medication by a doctor, and use of antidepressant or anxiolytic treatment before the attack. This was true regardless of the adjustment variables (exposure, sex, probable PTSD, or depression).

For the models adjusted for number of previous traumatic events and age, the results are provided in the supplementary material (see Table S2). The number of previous traumatic events is significant associated with a higher risk of self‐medication. Age is significantly and positively associated with self‐medication and the prescription of medication by a doctor.

## 4 Discussion

This study shows that help‐seeking is insufficient among the police staff three months after the attack. Only 36% of staff with probable PTSD or depression received specialist mental health care. Staff with partial or complete probable PTSD who were directly exposed to the attack were more likely to have taken psychotropic medication or received specialist mental health follow up than the rest of the police population surveyed. Police officers with symptoms of depression were more likely to have taken antidepressants before the attack.

In line with the second hypothesis, an increase in alcohol or tobacco consumption was observed among those directly exposed and those with probable PTSD. Increased use of tobacco or alcohol is associated with probable PTSD severity and mental health service use.

### 4.1 Healthcare Consumption

In accordance with the existing literature, the initiation of treatment or specialised post‐attack care was more frequent in police officers with probable PTSD than in those without PTSD in this study.

However, rates of seeking mental health care are low. In our study, the rate of specialist care among people with PTSD was 33% and the rate of general practitioner consultation was 72%. The use of health care by civilians in Strasbourg was also higher after the attack. The frequency of consulting a general practitioner was 34.6% among civilians without PTSD compared with 58.9% among civilians with probable PTSD, the frequency of consulting a mental health professional was 62% compared with 85.7%, and the use of psychotropic medication since the attack was 10% compared with 30% [2].

The following hypotheses may explain why police officers do not make sufficient use of mental healthcare: police culture; the stigma of being perceived as weak; and the fear of being deemed unfit for work or damaging one's career prospects [31]. Barriers experienced by the general population, such as a lack of awareness of mental health issues or the need for specialised care, are added to those specific to police staff [1].

Mental health stigma among police officers in the USA was observed in over 90% of officers in a 2021 study [4]. Soomro et al in 2019 demonstrated that police officers with PTSD have more negative attitudes and behaviours towards people with mental illness [32], which may exacerbate the challenges they face in accessing care in cases of mental distress.

A police staff may find it easier and more acceptable to consult a doctor about a physical problem. Berg et al. show that Norwegian police officers consult general practitioners, chiropractors, and physiotherapists more than the general population, and that they present their physical problems more than their psychological problems [5]. In line with this study, the consultation rate for general practitioners and physiotherapists was high in our study, with 62% and 24% of consultants respectively.

In our study, the use of health care by police staff with depression differs from that of police staff with probable PTSD. Police staff with depression had greater use of any type of care, consultation with a psychiatrist or psychologist (OR = 4.34 [2.08–9.03]), self‐medication (OR = 3.81 [2.11–6.90]), medication prescribed by a doctor (OR = 2.49 [1.39–4.44]), new psychotropic treatment since the attack (OR = 6. 90 [1.71–27.79]) and psychotropic treatment before the attack (6.03 [2.24–16.23]), while police staff with a PSTD only demonstrated higher consultation with a psychiatrist or psychologist (OR = 5.02 [2.44–10.32]) and new psychotropic treatment since the attack (OR = 4.59 [1.11–18.91]).

The use of health care for PTSD is in line with recommendations, with more counselling than psychotropic medication. To explain the use of care for depression, we can hypothesise the existence of three different profiles: (1) officers with a previous mental disorder who started or resumed previous treatment after the attack in the context of a depressive episode, (2) officers with previous symptoms who had not previously consulted a doctor and who used the attack as a reason to consult a doctor, or (3) people with de novo depression after the attack. The design of the study does not allow us to answer this question and further research is needed.

The models were adjusted for number of previous traumatic events and age. Only self‐medication was identified between a number of previous traumatic event and the use of healthcare services. One could hypothesize that previous events led police officers to believe that they could manage self‐medication on their own or that they may have been stigmatized in the past and therefore prefer self‐medication to mental health‐seeking.

### 4.2 Alcohol and Tobacco Consumption and Trends

The alcohol consumption in our population (36% at least at risk) is close to the figures reported in the literature review by Syed et al. in 2020, who estimated the prevalence of alcohol dependence at 5% and at‐risk consumption at 25.7%, giving a total of 30.7% [20].

In our study, police officers suffering from PTSD changed their alcohol and tobacco consumption to a greater extent (OR = 10.42 [3.45–31.43]) regardless of their exposure to the attack. In a study conducted in the USA, Dougherty et al. showed that excessive monthly alcohol consumption was associated with what he called “administrative stress” (stress related to the organisation, schedules and resources for police officers' administrative tasks) and with each additional critical incident reported by police officers [33]. They also found a link between improved resilience and reduced consumption.

Swat et al, examining the relationship between alcohol consumption and work‐related pressure among police officers, showed that their consumption was mediated by anxiety and depression [34].

The relationship between substance misuse and PTSD is complex. Lane et al published a review of the literature on this topic in 2019 [35]. They suggested several hypotheses, including self‐medication in the context of adaptive self‐efficacy. On self‐medication, Lane referred to the work of Khantzian, who hypothesised that low or moderate alcohol consumption would reduce the emotional numbness and detachment associated with PTSD, whereas higher levels of alcohol consumption would aim to reduce the intensity of emotions [36]. Alcohol would then be used to reduce the distress caused by PTSD symptoms. Normally, alcohol use is modulated by adaptive self‐efficacy to resist drinking. However, Alexander et al. showed that in the event of a disaster, sensitivity to adaptive self‐efficacy is reduced, leaving room for psychological distress and self‐medication [22].

In our study, police officers who are directly exposed are more likely to increase their use of tobacco or alcohol, in line with the results of Alexander et al [22]. We also found an increase in the consumption of tobacco or alcohol use with the severity of PTSD. In fact, for police staff with PTSD, increasing the use of tobacco or alcohol improves sleep immediately, but then the quality of sleep deteriorates. In the end, they consult a doctor with two medical problems: the PTSD and the misuse of substances. But we cannot rule out the possibility that the increased use of tobacco or alcohol is also a way of compensating for the lack of access to care.

The design of the study does not allow us to say whether it was the severity of the mental disorder, the lack of care, the combination, or some other reason that contributed to their use.

### 4.3 Improving Access to Mental Healthcare for Police Staff

Our results show the importance of improving access to mental healthcare for police staff. First, it is essential that police staff receive training in mental health first aid; several studies suggest that this would also be beneficial for their own mental health [9] by reducing stigma and thereby encouraging them to seek mental health support. Second, it is necessary to introduce systematic screening for mental health problems following large‐scale violent events. Third, mental health policies should also rely on prevention by striving to reduce work‐organisation related stress and improving management support and administration. Fourth to, further research is needed on the barriers to mental healthcare among French police staff to identify the most appropriate measures to be implemented.

One factor that has not been explored likely contributes to the lack of mental healthcare uptake among police officers in France. Indeed, the availability of mental healthcare in France is variable. Indeed, although police staff may benefit from psychological support by police psychologists, access to psychiatrists and psychotropic medication remain limited and externalised. Furthermore, although police psychologists are bound by professional confidentiality—since they are part of the police force—police officers may fear a lack of confidentiality and the potential repercussions of their confidences on their careers.

### 4.4 Strengths and Limitations

The main strength of our work is that it is the first French study to examine the use of mental health care and the consumption of alcohol and tobacco in a population made up only of police staff, and the first to look at this population after an attack. Studies concerning civilians are also far more numerous [3, 23] than those concerning professionals such as police officers [24].

Another positive aspect was the simultaneous collection of data on medical care, alcohol and tobacco use, and changes since the attack, as well as the search for probable PTSD, depression, and exposure level. This allowed us to compare consumption according to exposure and presence of probable PTSD or depression.

In terms of study design, the sudden onset of the event meant that we were unable to do prospective follow‐up and had to implement the research protocol within a limited timeframe. The unforeseen nature of the event makes it more difficult to use a before‐and‐after study methodology, and many studies only include victims. One of our strengths was therefore to be able to compare exposed and unexposed police staff.

The size of the sample may seem small, but it provides sufficient power to highlight interesting results in a singular context and with a specific population of police staff.

However, a larger sample size would have enabled us to analyse direct and indirect exposure separately. The various models were also constructed by classifying exposure into three categories, and the results were similar, apart from a loss of statistical power. The results are presented in the supplementary materials (Table S3).

The decision was made to analyse partial and complete PTSD together. This was done, not only due to the sample size, but also in reference to authors such as Bowler et al [37]. In his article on the follow‐up of police officers who responded to the attack on the World Trade Center, Bowler showed that some PTSD developed late, particularly among male police officers and concluded that it was therefore necessary to follow up all responders, whether or not they exhibited symptoms of PTSD over the long term.

There may be several biases, such as recruitment bias. Among police officers who respond, there may be an over‐representation of those who are anxious and at risk of mental health problems; among non‐respondents, there may be police officers who did not trust the anonymity of the questionnaire and did not respond for fear of stigma or career implications, resulting in an under‐representation. We compared respondents and non‐respondents based on key sociodemographic characteristics for the three largest departments (*N* = 1272). Respondents were 42.3 (8.8) years old, on average, compared to 40.6 (9.2) for non‐respondents (*p* = < 0.05). There was an overrepresentation of women (33.9% of respondents vs. 23.9% of non‐respondents) and police commissioners among respondents (2.7% of respondents vs. 0.4% of non‐respondents; *p* = < 0.05). As female gender is a risk factor for PTSD, the prevalence of PTSD in the population of police officers might have been overestimated in our study [38]. Declarative bias must be considered in this population, particularly for questions about alcohol consumption and changes in consumption, again due to fear of anonymity and stigma. To avoid too marked a memory bias, also linked to possible dissociative symptoms, we chose not to collect immediate care records.

The unpredictable nature of the event led us to propose a cross‐sectional study design. This design does not allow us to establish a causal link between the variables. Health surveillance‐type data collection, with regular standardised systematic medical monitoring, would provide data before and after an event of this type. It would also be useful to monitor the use of healthcare and substances such as alcohol and tobacco.

## 5 Conclusion

Our study shows that police officers with PTSD and depression sought specialist care for mental health problems after the Strasbourg attack, but at a lower rate than civilians. The type of care sought differed, with a lower proportion consulting a psychiatrist or psychologist and a higher proportion consulting a general practitioner. These findings may be explained by the fear of stigma and career repercussions among police officers, and by the self‐medication and self‐efficacy hypotheses. Alcohol use was similar to other police populations, but there was an increased use of alcohol and tobacco after attacks, which was associated with PTSD severity.

It is essential to improve the identification of staff with symptoms of PTSD, depression and addictive disorders and to encourage them to seek help or to follow up them. In addition, the barriers faced by French police officers in seeking mental health care need to be examined and resources found to reduce their fear of being stigmatised.

## Author Contributions

**Nathalie Nourry:** conceptualization, formal analysis, investigation, writing – original draft, writing – review and editing, visualization, project administration. **Ludivine Nohales:** writing – original draft, writing – review and editing. **Laurence Lalanne:** writing – review and editing. **Emmanuel Fort:** formal analysis, writing – review and editing, visualization. **Maria Gonzalez:** writing – review and editing. **Pierre Vidailhet:** conceptualization, writing – review and editing. **Amaury C. Mengin:** conceptualization, writing – review and editing. **Barbara Charbotel:** writing – review and editing, supervision.

## Ethics Statement

The study was approved by the Comité d'Ethique pour la Recherche, the Ethics Committee of the Faculties of Medicine, Odontology, Pharmacy, Nursing, Physiotherapy and Maieutics, and the University Hospitals of Strasbourg, France (CE‐2019‐17). Prior to participation, all subjects received written information detailing the objectives of the study. Written consent was obtained from all participants. The anonymised data collected for this research study are not linked to any personally identifiable information.

## Conflicts of Interest

The authors declare no conflicts of interest.

## Supporting information

Supporting File 1

Supporting File 2

Supporting File 3

## Data Availability

The data that support the findings of this study are available on request from the corresponding author. The data are not publicly available due to privacy or ethical restrictions. According to European law (GDPR) data containing potentially identifying or sensitive patient information are restricted; our data involving clinical participants are not freely available in a public repository. However, data are available on request from corresponding author.
