# Supplementary material for: Help‐Seeking and Substance Use Among Police Staff After the 2018 Strasbourg Christmas Market Attack
Source: Am J Ind Med. 2026 May 24;69(8):601–13. doi: 10.1002/ajim.70092 (PMC13350426; doi:10.1002/ajim.70092)
Supplement: Supplementary file 1 — Supporting File 1 [file AJIM-69-601-s001.docx]

Table SI. Questions used to assess exposure degree among police personnel

|  | **Have you been directly or indirectly involved in any of the following events?** | yes | no | Category if answered “yes” |
| --- | --- | --- | --- | --- |
| 1 | Procedure(s) prior to the day of the attack and concerning the terrorist |  |  | Not exposed |
| 2 | Police intervention at the terrorist's home on the morning of the attacks |  |  | Not exposed |
| 3 | Attack in the city centre of Strasbourg (intervention on the spot) |  |  | Direct exposure |
| 4 | Pursuit of the terrorist as he was fleeing the city centre |  |  | Direct exposure |
| 5 | Search for the terrorist in or outside Strasbourg |  |  | Indirect exposure |
| 6 | Neutralization of the terrorist |  |  | Direct exposure |
| 7 | I have not been involved in any of these situations |  |  | Not exposed |
|  | **The following questions will help us to better describe the circumstances in which you were involved.** |  |  |  |
| 8 | Did you make eye contact with anyone directly threatened or injured by the terrorist? |  |  | Indirect exposure |
| 9 | Have you been in contact by telephone with anyone directly threatened or injured by the terrorist? |  |  | Indirect exposure |
| 10 | Have you performed a rescue action on a victim (injured or dead person)? |  |  | Direct exposure |
| 11 | Did you provide psychosocial support (comfort) to a victim (injured/hostage) or a relative of a victim (injured/hostage/deceased) |  |  | Indirect exposure |
| 12 | Did you visit the scene of the attack (Strasbourg city centre) |  |  | Direct exposure |
| 13 | Were you present at the terrorist's neutralization on Thursday night? |  |  | Direct exposure |
| 14 | Did you have any contact with the terrorist (visual/auditory, excluding detonations related to assaults by intervention forces) |  |  | Direct exposure |
| 15 | I have not experienced any of these situations |  |  | Not exposed |

*Legend: As soon as a participant answered “yes” to any question corresponding to a category of exposure, he/she was attributed to the most important degree of exposure. For example: if a participant answered “yes” to the question 2 and 3, he/she was classified as “Direct exposure”*
