# Supplementary material for: Help‐Seeking and Substance Use Among Police Staff After the 2018 Strasbourg Christmas Market Attack
Source: Am J Ind Med. 2026 May 24;69(8):601–13. doi: 10.1002/ajim.70092 (PMC13350426; doi:10.1002/ajim.70092)
Supplement: Supplementary file 2 — Supporting File 2 [file AJIM-69-601-s003.docx]

**Table S II Results of multivariable analysis of healthcare, alcohol and tobacco use and trends, adjusted for age and number of previous of traumatic events**

|  | Independant variable | Models adjusted for probable ptsd and other variables ^1^ | Models adjusted for depression and other variables ^1^ |
| --- | --- | --- | --- |
| ! General practitionner | Exposure *ref = no +Expo indirect*  *Expo direct* | 1  0.90 [0.60-1.36] | 1  0.94 [0.63-1.14] |
|  | Gender *ref = male* | **1**  **1.80 [1.13-2.86]** | **1**  **1.83 [1.15-2.90]** |
|  | Probable PTSD  *ref= no PTSD* | 1  1.47 [0.78-2.78] |  |
|  | Depression   *ref = no* |  | 1  1.27 [0.67-2.41] |
|  | Number of previous traumatic event | 1.03 [0.98-1.07] | 1.03 [0.98-1.07] |
|  | Age | 1.01 [0.99-1.03] | 1.01 [0.99-1.03] |
| Physiotherapist | Exposure *ref = no +Expo indirect*  *Expo direct* | 1  1.22 [0.78-1.93] | 1  1.27 [0.81-1.98] |
|  | Gender *ref = male* | 1  1.25 [0.76-2.04] | 1  1.22 [0.74-2.01] |
|  | Probable PTSD  *ref= no PTSD* | 1  1.27 [0.68-2.39] |  |
|  | Depression *ref = no* |  | 1  1.40 [0.74-2.64] |
|  | Number of previous traumatic event | 1.05 [0.99-1.11] | 1.05 [0.99-1.11] |
|  | Age | 1.001 [0.98-1.03] | 1.01 [0.98-1.03] |
| consultation with a psychiatrist or psychologist | Exposure  *ref = no +Expo indirecte*  *Expo directe* | **1**  **2.35 [1.14-4.85]** | **1**  **3.36 [1.62 6.94]** |
|  | Gender *ref = male* | **1**  **4.43 [2.18-9.00]** | **1**  **4.65 [2.29-9.44** |
|  | Probable PTSD  *ref= no PTSD* | **1**  **4.75 [2.29-9.86]** |  |
|  | Depression *ref = no* |  | **1**  **4.21 [1.98-8.94]** |
|  | Number of previous traumatic event | 1.02 [0.94-1.11] | 1.02 [0.94-1.11] |
|  | Age | 0.99 [0.95-1.02] | 0.98 [0.94-1.02] |
| self-medication | Exposure *ref = no +Expo indirect*  *Expo direct* | 1  0.77 [0.45-1.15] | 1  0.75 [0.47-1.99] |
|  | Gender *ref = male* | **1**  **2.59 [1.62-4.13]** | **1**  **2.26 [1.40-3.64]** |
|  | Probable PTSD  *ref= no PTSD* | 1  1.41 [0.75-2.62] |  |
|  | Depression *ref = no* |  | **1**  **3.55 [1.92-6.57]** |
|  | Number of previous traumatic event | **1.08 [1.03-1.14]** | **1.07 [1.02-1.13]** |
|  | Age | **1.04 [1.01-1.06]** | **1.04 [1.01-1.07]** |
| Prescription drugs | Exposure *ref = no +Expo indirect*  *Expo direct* | 1  1.04 [0.67-1.60] | 1  1.05 [0.68-1.61] |
|  | Gender *ref = male* | **1**  **1.97 [1.25-3.10]** | **1**  **1.69 [1.07-2.68]** |
|  | Probable PTSD  *ref= no PTSD* | 1  0.98 [0.53-1.82] |  |
|  | Depression  *ref = no* |  | 1  **2.67 [1.46-4.87]** |
|  | Number of previous traumatic event | 1.02 [0.97-1.07] | 1.01 [0.96-1.05] |
|  | Age | **1.06 [1.03-1.08]** | **1.06 [1.03-1.08]** |
| New “psy” treatment since attack  oui n=9 | Exposure *ref = no +Expo indirect*  *Expo direct* | 1  4.16 [0.79-21.96] | **1**  **5.65 [1.06-30.23]** |
|  | Gender *ref = male* | 1  0.81 [0.15-4.32] | 1  0.82 [0.15-4.50] |
|  | Probable PTSD  *ref= no PTSD* | **1**  **4.37 [1.04-18.47]** |  |
|  | Depression ref = no |  | **1**  **6.66 [1.60-27.70]** |
|  | Number of previous traumatic event | 1.03 [0.86-1.23] | 1.01 [0.84-1.22] |
|  | Age | **0.99 [0.92-1.08]** | 0.99 [0.91-1.08] |
| treatment started before the attack | Exposure *ref = no +Expo indirect*  *Expo direct e* | 1  0.65 [0.21-2.00] | 1  0.77 [0.25-2.40] |
|  | Gender *ref = male* | **1**  **3.64 [1.36-9.76]** | 1  2.56 [0.91-7.21] |
|  | Probable PTSD  *ref= no PTSD* | 1  1.73 [0.51-5.89] |  |
|  | Depression ref = no |  | **1**  **6.39 [2.23-18.24]** |
|  | Number of previous traumatic event | 1.04 [0.93-1.16] | 1.00 [0.88-1.12] |
|  | Age | 1.04 [0.99-1.09] | 1.04 [0.98-1.10] |
| **Modif alcool tabac 2cl** | Exposure *ref = no +Expo indirect*  *Expo direct* | **1**  **3.53 [1.02-12.25]** | **1**  **5.29 [1.57-17.84]** |
|  | Gender *ref = male* | 1.71 [0.53-5.55] | 1  2.45 [.080-7.51] |
|  | Probable PTSD  *ref= no PTSD* | **1**  **9.43 [3.07-28.95]** |  |
|  | Depression ref = no |  | 1  2.03 [.58-7.05] |
|  | Number of previous traumatic event | 1.13 [0.99-1.32] | 1.15 [.99-1.33] |
|  | Age | 1.01 [0.95-1.08] | 0.99 [0.94-1.06] |

^1^ Exposure, gender, number of previous of traumatic event, age
