# Supplementary material for: Help‐Seeking and Substance Use Among Police Staff After the 2018 Strasbourg Christmas Market Attack
Source: Am J Ind Med. 2026 May 24;69(8):601–13. doi: 10.1002/ajim.70092 (PMC13350426; doi:10.1002/ajim.70092)
Supplement: Supplementary file 3 — Supporting File 3 [file AJIM-69-601-s002.docx]

Table S III Results of multivariable analysis of healthcare, alcohol and tobacco use and trends with exposure with 3 classes, adjusted for age and number previous traumatic events

|  | Independant variable | Models adjusted for probable PTSD and other variables ^1^ | Models adjusted for depression and other variables ^1^ |
| --- | --- | --- | --- |
| General practitionner | Exposure *ref = no*  *Expo indirect*  *Expo direct* | 1  0.99 [0.57-1.72]  0.90 [0.58-1.40] | 1   - 1. [0.58-1.74]   0.94 [0.61-1.46 |
|  | Gender *ref = male* | **1**  **1.80 [1.13-2.86]** | **1**  **1.83 [1.15-2.91]** |
|  | Probable PTSD  *ref= no PTSD* | 1  1.47 [0.78-2.79] |  |
|  | Depression  *ref = no* |  | 1  1.27 [0.67-2.41] |
|  | Number of previous of traumatic event | 1.03 [0.98-1.07] | 1.03 [0.98-1.07] |
|  | Age | 1.01 [0.99-1.03] | 1.01 [0.99-1.03] |
| Physiotherapist | Exposure *ref = no*  *Expo indirect*  *Expo direct* | 1  1.17 [0.63-2.17]  1.29 [0.78-2.12] | 1  1.17 [0.63-2.18]  1.33 [0.81-2.18] |
|  | Gender *ref = male* | 1  1.26 [0.77-1.06] | 1  1.23 [0.75-2.03] |
|  | Probable PTSD  *ref= no PTSD* | 1  1.26 [0.67-2.37] |  |
|  | Depression  *ref = no* |  | 1  1.39 [0.74-2.63] |
|  | Number of previous of traumatic event | 1.05 [0.99-1.11] | 1.05 [0.99-1.10] |
|  | Age | 1.01 [0.98-1.03] | 1.00 [0.98-1.03] |
| consultation with a psychiatrist or psychologist | Exposure *ref = no*  *Expo indirect*  *Expo direct* | 1  1.12 [0.38-3.31]  **2.43 [1.08-5.47]** | 1  1.27 [0.43-3.77]  **3.62 [1.61-8.15]** |
|  | Gender *ref = male* | **1**  **4.44 [2.18-9.04]** | **1**  **4.69 [2.31-9.53]** |
|  | Probable PTSD  *ref= no PTSD* | 1  **4.72 [2.27-9.83]** |  |
|  | Depression *ref = no* |  | 1  **4.21 [1.98-8.95]** |
|  | Number of previous of traumatic event | 1.02 [0.94-1.11] | 1.01 [0.93-1.11] |
|  | Age | 0.99 [0.95-1.02] | 0.98 [0.94-1.02] |
| self-medication | Exposure *ref = no*  *Expo indirect*  *Expo direct* | 1  0.87 [0.48-1.58]  0.69 [0.42-1.14] | 1  0.85 [0.46-1.57]  0.72 [0.44-1.18] |
|  | Gender *ref = male* | **1**  **2.57 [1.61-4.10]** | **1**  **2.25 [1.39-3.62]** |
|  | Probable PTSD  *ref= no PTSD* | 1  1.42 [0.76-2.65] |  |
|  | Depression *ref = no* |  | **1**  **3.57 [1.93-6.60]** |
|  | Number of previous of traumatic event | **1.09 [1.03-1.14]** | **1.07 [1.02-1.13]** |
|  | Age | **1.04 [1.01-1.06]** | **1.04 [1.01-1.07]** |
| Prescription drugs | Exposure *ref = no*  *Expo indirect*  *Expo direct* | 1  0.69 [0.38-1.25]  0.93 [0.58-1.48] | 1  0.67 [0.37-1.22]  0.93 [0.58-1.48] |
|  | Gender *ref = male* | **1**  **1.94 [1.23-3.05]** | **1**  **1.67 [1.05-2.65]** |
|  | Probable PTSD  *ref= no PTSD* | 1  1.00 [0.54-1.87] |  |
|  | Depression *ref = no* |  | **1**  **2.71 [1.18-4.96]** |
|  | Number of previous of traumatic event | 1.02 [0.98-1.07] | 1.01 [0.96-1.06] |
|  | Age | **1.06 [1.03-1.08]** | **1.06 [1.03-1.08]** |
| New “psy” treatment since attack  oui n=9 | Exposure *ref = no*  *Expo indirect*  *Expo direct* | 1  2.26 [0.13-38.07]  5.87 [0.65-52.91] | 1  2.43 [0.14-41.73]  8.18 [0.89-75.24] |
|  | Gender *ref = male* | 1  0.82 [0.15-4.39] | 1  0.85 [0.16-4.06] |
|  | Probable PTSD  *ref= no PTSD* | **1**  **4.28 [1.01-18.10]** |  |
|  | Depression *ref = no* |  | **1**  **6.64 [1.58-27.84]** |
|  | Number of previous of traumatic event | 1.02 [0.85-1.23] | 1.00 [0.83-1.21] |
|  | Age | 0.99 [0.91-1.08] | 0.99 [0.91-1.08] |
| treatment started before the attack | Exposure *ref = no*  *Expo indirect*  *Expo direct e* | 1  0.67 [0.17-2.61]  0.58 [0.18-1.89] | 1  0.71 0.17-2.89]  0.69 [0.21-2.32] |
|  | Gender *ref = male* | **1**  **3.60 [1.34-9.66]** | 1  2.51 [0.89-7.13] |
|  | Probable PTSD  *ref= no PTSD* | 1  1.78 [0.52-6.09] |  |
|  | Depression *ref = no* |  | **1**  **6.35 [2.23-18.11]** |
|  | Number of previous of traumatic event | 1.04 [0.93-1.17] | 1.00 [0.89-1.14] |
|  | Age | 1.04 [0.99-1.09] | 1.04 [0.98-1.10] |
| **Modif alcool tabac 2cl** | Exposure *ref = no*  *Expo indirect*  *Expo direct* | 1  0.48 [0.45-5.17]  2.77 [0.68-11.31] | 1  0.67 [0.07-6.79]  **4.71 [1.21-18.23]** |
|  | Gender *ref = male* | 1  1.71 [0.52-5.55] | 1  2.44 [0.79-7.51] |
|  | Probable PTSD  *ref= no PTSD* | **1**  **9.77 [3.16-30.22]** |  |
|  | Depression *ref = no* |  | 1  2.05 [0.59-7.12] |
|  | Number of previous of traumatic event | 1.14 [0.97-1.33] | 1.15 [0.99-1.33] |
|  | Age | 1.01 [0.95-1.08] | 1.00 [0.94-1.06] |

^1^ Exposure, Gender, number of previous traumatic event, age
